# Intractable hiccups after VNS implantation: a case report

**DOI:** 10.1186/s12883-023-03352-x

**Published:** 2023-08-10

**Authors:** Susan Zhang Recio, Myriam Abdennadher

**Affiliations:** Department of Neurology, Boston Medical Center/Boston University Chobanian & Avedisian School of Medicine, Boston, MA 02118 USA

**Keywords:** Intractable hiccups, Vagus nerve stimulator, Singultus, Neuromodulation, Epilepsy, Case report

## Abstract

**Background:**

Hiccups (medically termed, “singultus”), when intractable, can cause significant medical consequences such as aspiration, malnutrition, and depression, leading to poor quality of life. Several case reports have shown that vagus nerve stimulator (VNS) implantation can help treat central idiopathic intractable hiccups. However, we present a contrary case of a patient who developed intractable singultus following VNS placement for medically refractory epilepsy.

**Case presentation:**

We report a 71-year-old male patient with drug-resistant epilepsy who underwent VNS implantation and developed intractable hiccups shortly thereafter. The hiccups were severe and persistent, such that the patient developed a Mallory-Weiss tear, which required intensive care, invasive intubation and mechanical ventilation, and a prolonged rehabilitation course. Despite multiple therapies including phrenic nerve block and Nissen fundoplication, the patient’s hiccups persisted and only stopped once the VNS was permanently deactivated.

**Conclusions:**

Little is known about the incidence of hiccups after VNS implantation. We present one case of hiccups as a direct consequence of VNS implantation. The clinical impact of this report is significant given the relative unfamiliarity of hiccups as an adverse effect of VNS implantation. Neurologists and epileptologists, who present VNS implantation as a surgical option for seizure control to their patients, should be aware of the possibility of singultus development and its significant physical and emotional ramifications.

## Background

Hiccups (medically termed, “singultus”) are often benign and self-limiting, but in rare cases they can be intractable and a sign of underlying pathology. Intractable singultus is defined as hiccups persisting for > 1 month [1–3]. Table 1 lists some of the causes of persistent and refractory hiccups that are commonly cited in the literature [4]. Intractable hiccups can result in significant and life-threatening medical consequences including insomnia, depression, malnutrition/anorexia, aspiration, pneumonia, and impaired wound healing [5].

**Table 1:** Causes of persistent and intractable hiccups

**Cardiovascular disorders**	**Central nervous system disorders**	**Drugs**	**Ear/Nose/ Throat Disorders**	**Infectious Disorders**	**Intrathoracic Disorders**	**Gastrointestinal Disorders**	**Metabolic & endocrine disorders**	**Psychogenic disorders** [26]	**Surgery**
Atrial pacing Aortic aneurysm (thoracic or abdominal) Catheter ablation of atrial fibrillation Myocardial infarction Pericarditis Temporal arteritis	Aneurysms (posterior inferior cerebellar artery) Encephalitis Lateral medullary syndrome [27] Meningitis Demyelinating disorders (multiple sclerosis, neuromyelitis optica [28]) Neoplasms (astrocytoma, brain stem tumor) Parkinson disease Seizure Stroke Syringomyelia Vascular malformations (cavernoma) [29]	Alpha-methyldopa Aripiprazole Azithromycin Benzodiazepines (diazepam, midazolam) Chemotherapy (carboplatin, cisplatin [30], etoposide, fluorouracil, irinotecan, levofolinate, oxaliplatin) Dexamethasone Donepezil Ethanol Levodopa Methohexital Morphine Pergolide Piribedil Sulfonamides Tramadol	Cough Foreign body irritation of tympanic membrane (hair) Goiter Laryngitis Neck cyst Neoplasm Pharyngitis Recent intubation	*Helicobacter pylori* Herpes simplex virus Herpes zoster virus Influenza Malaria Neurosyphilis Tuberculosis	Asthma Bronchitis Diaphragmatic tumor or a hernia Empyema Lymphadenopathy Mediastinitis Neoplasms Pleuritis Pneumonia Pulmonary embolus	Aerophagia Bowel obstruction Gastric distention Esophageal cancer [31] Esophagitis (infectious or erosive) [32] Gallbladder disease Gastric distension Gastro-esophageal reflux disease (GERD) [33] Hepatitis Neoplasms Pancreatitis Peptic ulcer disease Stomach volvulus Subphrenic abscess	Hypocapnia Hypocalcemia Hypokalemia Hyponatremia Diabetes mellitus Uremia	Anxiety, Excitation [13] Stress Hyperventilation Malingering Somatization Conversion reaction (34)	Anesthesia (barbiturates, bupivacaine epidural, isoflurane, methohexital, propofol) Bronchoscopy Gastric insufflation during endoscopy Post-operative [35] Tracheostomy Sedation during endoscopy or colonoscopy [36]

**Fig. 1:**
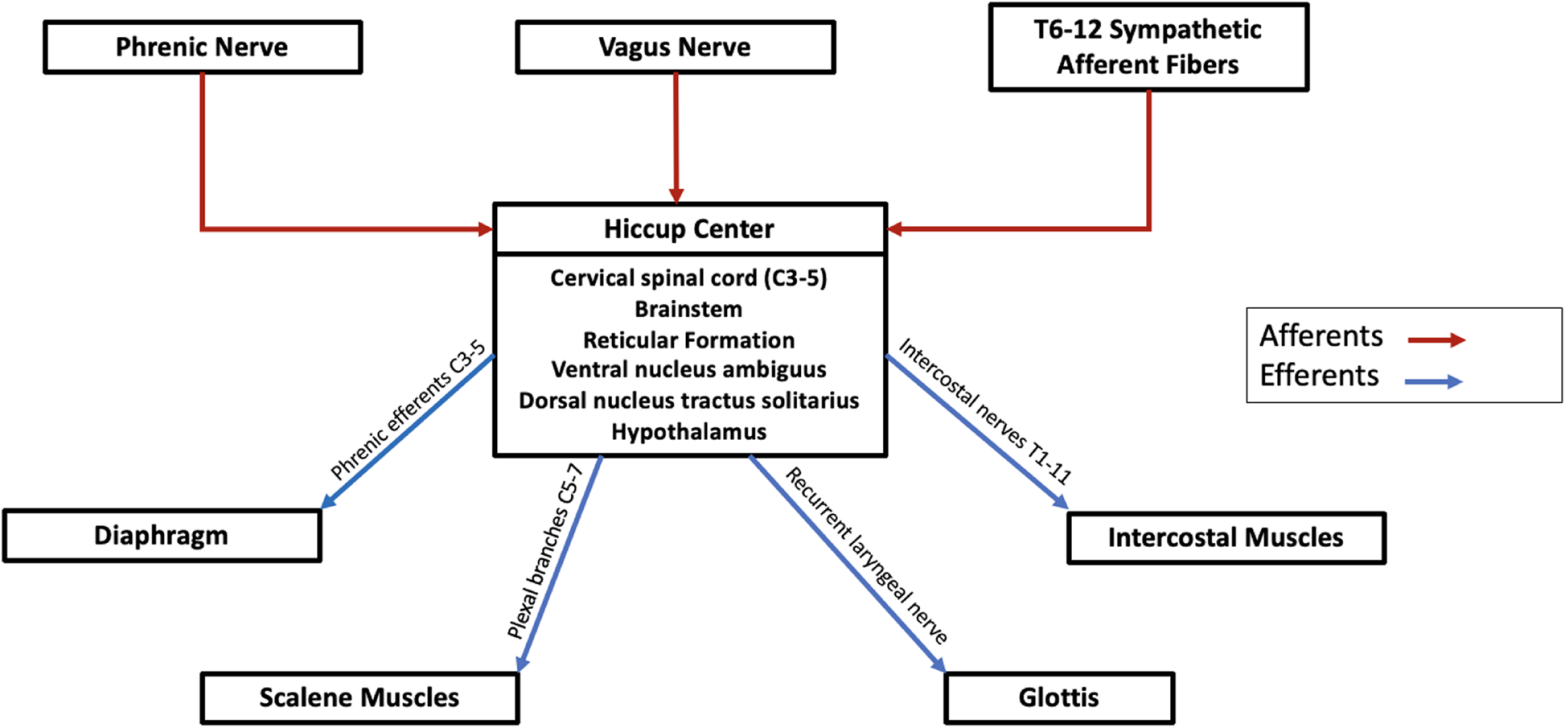
Pathophysiology of singultus. Central, afferent, and efferent pathways of the hiccup reflex.

The pathophysiology of singultus is complex and Fig. 1 depicts the involved anatomy and overall pathway. The hiccup reflex arc has afferent, central, and efferent components. The afferent limb is composed of the vagus and phrenic nerves, as well as T6-12 sympathetic fibers. The efferent limb consists of the diaphragm (innervated by the phrenic nerve C3-5), scalene muscles (innervated by plexal branches C5-7), glottis (innervated by the recurrent laryngeal nerve), and intercostal muscles (innervated by intercostal nerves T1-11) [1, 2, 6]. The central hiccup center is distributed over spinal cord segments rostral to the medulla (C3-5) in the reticular formation, the Pre-Botzinger complex and nucleus tractus solitarius in the brainstem, the hypothalamus, and the mesial temporal lobes [1, 2, 6–8].

The vagus nerve (also known as the “wandering nerve” or “vagabond nerve”) innervates multiple organs to regulate autonomic functions of the cardiovascular, respiratory, and gastrointestinal systems [9, 10]. There are several case reports in the literature that show benefits of vagal nerve stimulation to treat central idiopathic intractable hiccups [1, 6, 11]. This is usually achieved through vagus nerve stimulator (VNS) implantation, typically used for medically refractory epilepsy. Vagal nerve stimulation is not currently approved by the Food and Drug Administration (FDA) as a therapy for intractable hiccups, and the data is mixed regarding its efficacy to treat singultus [1, 11]. Nevertheless, outcomes to date are either positive with partial or short-term hiccup resolution, or equivocal with no significant benefit from vagal nerve stimulation [11].

We present a case of intractable singultus after VNS placement in a patient with refractory epilepsy whose hiccups resolved only with VNS deactivation. The incidence of hiccups after VNS implantation is unclear and much remains to be done to understand this relationship. This case is a significant addition to the literature given that multiple prior studies have reported the opposite association of VNS placement as a potential treatment for intractable hiccups rather than its apparent cause.

## Case presentation

A 71-year-old male presented to the clinic with epilepsy, first diagnosed at the age of 2 years. For many years, his seizures were controlled with medication. In the second decade of life, the patient sustained a severe fall leading to traumatic brain injury confirmed on brain magnetic resonance imaging (MRI), which demonstrated left frontal encephalomalacia. Shortly thereafter, seizures recurred and failed to respond to multiple anti-seizure medications including levetiracetam (2500 mg/day), phenytoin (350 mg/day, then lowered to 300 mg/day), lacosamide (300 mg/day), and topiramate (400 mg/day). The patient’s seizure semiology included focal impaired awareness seizures (up to 5 per month) and bilateral tonic clonic seizures (1 to 2 per year). The patient eventually underwent surgery with left-sided VNS implantation (lead wire wrapped around the left vagus nerve, generator implanted on the left chest wall) that, unfortunately, did not reduce his seizure burden. Furthermore, he subsequently developed hiccups that persisted despite medical treatment, resulting in a Mallory-Weiss tear requiring intensive care unit (ICU) admission with intubation and ventilation followed by a month-long rehabilitation. The patient then underwent a Nissen fundoplication followed by two phrenic nerve blocks that led to only transient hiccup resolution for 13 months. Finally, the VNS was turned off, which was followed by complete hiccup resolution as of the most recent follow-up appointments at the time of this publication. The patient’s seizures also resolved at the age of 54 after clobazam (30 mg/day) was added to his anti-seizure medication regimen.

## Discussion and conclusions

Hiccups can be attributed to almost 4000 hospitalizations per year in the United States [5, 12].

When severe and intractable, they have a devastating impact on one’s physical and mental health. Multiple peripheral and central etiologies have been identified for intractable singultus [5, 13, 14], but vagal nerve stimulation has never been among them; rather, several cases have highlighted VNS implantation as a potential therapy for hiccups [1, 11].

VNS implantation was FDA-approved as an adjuvant therapy for medically refractory epilepsy in 1997 [9, 15]. It is overall well-tolerated with relatively few side effects, the most common of which are laryngeal (e.g. hoarseness, dysphonia) due to the vagus nerve’s effect on vocal cord motion and supraglottic muscle tension [16]. Other side effects include bradyarrhythmias (from parasympathetic stimulation of the atrioventricular node) [9], cardiac syncope and asystole [17], respiratory problems (cough, dyspnea, sleep disordered breathing) [18, 19], surgical or hardware complications (infection, lead malfunction, vocal cord palsy) [20], Horner syndrome [21], and dysphagia and/or aspiration [22]. However, intractable singultus has not previously been recognized as a potential VNS complication to the best knowledge of the authors.

The exact mechanism of how hiccups are triggered is unknown, although there are likely multiple ways to stimulate the reflex arc given its wide distribution via the autonomic nervous system as outlined in Fig. 1. Any process that irritates or damages part of the hiccup reflex or the autonomic nervous system including the vagus and phrenic nerves can lead to singultus [1]. We hypothesize that in our patient, the VNS lead to uninhibited vagal nerve firing through direct electrical pulses from the lead wire wrapped around the nerve, causing refractory singultus. However, more research is needed to understand why in some cases this electrical stimulus can lead to hiccup resolution, whereas in our patient, it triggered new onset hiccups.

Hiccups can also be seen in strokes of the brainstem [23] and it has been shown that vagal nerve stimulation modulates central parasympathetic activity that may lead to post-stimulus brainstem plasticity [24]. Given that the vagus nerve nucleus lies within the nucleus ambiguus in the brainstem, this may be another potential mechanism for singultus. Additionally, persistent hiccups have been reported in demyelinating disorders such as Neuromyelitis Optica Spectrum Disorder (NMOSD) that tend to involve the area postrema [25]. Inflammation of this region can lead to singultus as well as nausea and vomiting because it functions as an emetic reflex center [25]. We consider this a less likely etiology of hiccups in our patient given his clinical history, demographics, lack of enhancement on prior brain MRI with contrast, immediate hiccup resolution following VNS removal, and no known steroid treatment. However, given that cerebrospinal fluid (CSF) studies and demyelinating disease markers such as aquaporin-4 antibody were not sent on our patient, it cannot be ruled out completely.

Intractable singultus can be incredibly distressing for patients and negatively impact their health and quality of life. We present a case report of hiccups as a direct consequence of VNS implantation, an established therapy for medically refractory epilepsy and an off-label treatment for intractable singultus in several prior case reports. Our results demonstrate that the relationship between vagal nerve stimulation and hiccups remains to be further clarified. Providers recommending or prescribing VNS implantation for seizures should be aware of hiccups as a rare but possible side effect, and those considering VNS implantation for the treatment of singultus should exercise caution that this may trigger or worsen the hiccups. Additional research is merited to better understand the complex phenomenon of hiccups and how vagal nerve stimulation modulates its pathogenesis.

## Data Availability

Not applicable.

## Abbreviations

VNS: Vagus nerve stimulator
FDA: Food and Drug Administration
MRI: Magnetic resonance imaging
ICU: Intensive care unit
NMOSD: Neuromyelitis Optica Spectrum Disorder
CSF: Cerebrospinal fluid
